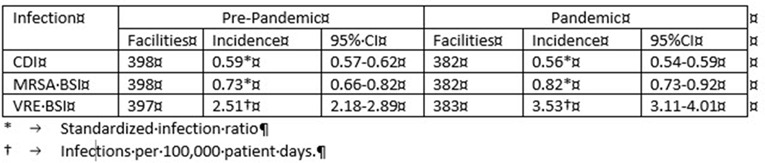# Comparing Hospital Healthcare-Associated Infection Incidence During Pre-COVID-19 Pandemic and Pandemic Eras

**DOI:** 10.1017/ash.2021.84

**Published:** 2021-07-29

**Authors:** Andrea Parriott, N. Neely Kazerouni, Erin Epson

## Abstract

**Background:** Diversion of resources from infection prevention activities, personal protective equipment supply shortages, conservation (extended use and reuse) or overuse with multiple gown and glove layers, and antimicrobial prescribing changes during the COVID-19 pandemic might increase healthcare-associated infection (HAI) incidence and antimicrobial resistance. We compared the incidences of *Clostridioides difficile* infection (CDI), methicillin-resistant *Staphyloccocus aureus* bloodstream infection (MRSA BSI), and vancomycin-resistant enterococci bloodstream infection (VRE BSI) reported by California hospitals during the COVID-19 pandemic with incidence data collected prior to the pandemic. **Methods:** Using data reported by hospitals to the California Department of Health via the NHSN, we compared incidences in the second and third quarters of 2020 (pandemic) to the second and third quarters of 2019 (before the pandemic). For CDI and MRSA BSI, we compared the standardized infection ratios (SIRs, based on the 2015 national baseline), and we calculated the *P* values. No adjustment model is available for VRE BSI; thus, we measured incidence via crude incidence rates (infections per 100,000 patient days). We calculated incidence rate ratio (IRR) with 95% CI for VRE BSI. To examine the possible effect of missing data during the pandemic, we performed a sensitivity analysis by excluding all facilities that had incomplete data reporting at any time during either analysis period. **Results:** Incidence measures and numbers of facilities contributing data in prepandemic and pandemic periods are shown in Table 1. There were no statistically significant changes in SIRs at *P* = .05 for either MRSA BSI or CDI between the prepandemic and pandemic periods (MRSA BSI *P* = .17; CDI *P* = .08). Crude VRE BSI incidence increased during the pandemic compared to the prepandemic period (IRR, 1.40; 95% CI, 1.16–1.70). Excluding facilities with incomplete data had minimal effect. **Conclusions:** We found insufficient evidence that MRSA BSI or CDI incidence changed in California hospitals during the pandemic relative to the prepandemic period; however, there was a significant increase in the crude incidence of VRE BSI. Next, we will include interrupted time series analyses to assess departure from long-term trends, including a risk-adjusted model for VRE BSI. Additionally, we will evaluate for changes in central-line–associated bloodstream infection incidence and antimicrobial resistance among HAI pathogens.

**Funding:** No

**Disclosures:** None

**Table 1.**